# SP140L, an Evolutionarily Recent Member of the SP100 Family, Is an Autoantigen in Primary Biliary Cirrhosis

**DOI:** 10.1155/2015/526518

**Published:** 2015-08-11

**Authors:** Mario Saare, Uku Hämarik, Rainis Venta, Marina Panarina, Chiara Zucchelli, Maire Pihlap, Anu Remm, Kai Kisand, Urve Toots, Kaidi Möll, Riina Salupere, Giovanna Musco, Raivo Uibo, Pärt Peterson

**Affiliations:** ^1^Molecular Pathology Group, Institute of Biomedicine and Translational Medicine, University of Tartu, 50411 Tartu, Estonia; ^2^Institute of Technology, University of Tartu, 50411 Tartu, Estonia; ^3^Department of Immunology, Institute of Biomedicine and Translational Medicine, University of Tartu, 50411 Tartu, Estonia; ^4^Biomolecular NMR Laboratory, Dulbecco Telethon Institute c/o S. Raffaele Scientific Institute, 20132 Milan, Italy; ^5^Icosagen AS, 61713 Tartumaa, Estonia; ^6^Department of Internal Medicine, Tartu University Hospital, 51014 Tartu, Estonia

## Abstract

The SP100 family members comprise a set of closely related genes on chromosome 2q37.1. The widely expressed SP100 and the leukocyte-specific proteins SP110 and SP140 have been associated with transcriptional regulation and various human diseases. Here, we have characterized the SP100 family member SP140L. The genome sequence analysis showed the formation of *SP140L* gene through rearrangements of the two neighboring genes, *SP100* and *SP140*, during the evolution of higher primates. The *SP140L* expression is interferon-inducible with high transcript levels in B cells and other peripheral blood mononuclear cells. Subcellularly, SP140L colocalizes with SP100 and SP140 in nuclear structures that are devoid of SP110, PML, or p300 proteins. Similarly to SP100 and SP140 protein, we detected serum autoantibodies to SP140L in patients with primary biliary cirrhosis using luciferase immunoprecipitation system and immunoblotting assays. In conclusion, our results show that SP140L is phylogenetically recent member of SP100 proteins and acts as an autoantigen in primary biliary cirrhosis patients.

## 1. Introduction

The SP100 family genes* SP100*,* SP110*, and* SP140* encode highly similar proteins that are mainly expressed in leukocytes [1–3], and perturbations of those genes have been associated with human cancers and immune diseases. Single nucleotide polymorphisms in* SP140* gene have been correlated with lower expression of SP140 together with higher incidence of chronic lymphocytic leukemia [4] and multiple myeloma [5]. Polymorphisms in the* SP140* gene are also linked with risk for multiple sclerosis [6] and Crohn's disease [7]. Mutations in the* SP110* gene result in venoocclusive disease with immunodeficiency; this disease is an autosomal recessive disorder of severe combined T and B cell immunodeficiency with absent lymph node germinal centers [8]. In addition, SP100 and SP140 are autoantigenic targets in primary biliary cirrhosis (PBC) [9, 10], a slowly progressing autoimmune disease that destroys primarily the bile canaliculi and leads to cholestasis [11].

Interferons (IFNs) and other viral infection-related stimuli strongly enhance SP100 family gene expression [12–16]. Links with viral machinery are also reflected in many direct interactions between viral and SP100 family proteins. SP140 was reported as an interaction partner of human immunodeficiency virus Vif protein in a yeast two-hybrid screen and further shown to partially disperse into the cytosol as a consequence of this interaction [17], although the functional significance of this process has not been elucidated. Of note, the SP100 isoform A interacts directly through its homogenously staining region (HSR) with the Epstein-Barr virus nuclear antigen leader protein and is a major mediator B cell immortalization caused by Epstein-Barr virus (EBV) [18]. Longer isoforms of SP100 have also been shown to repress the expression of proteins needed for the initiation of herpes simplex virus 1 lytic infection [19], and SUMOylated forms of SP100 are degraded via ubiquitination by viral proteins during the initiation [20]. Furthermore, SP110 interacts with an EBV early replicative cycle protein to increase the level of EBV lytic transcripts [21].

The SP100 family members share common domains, such as the N-terminal HSR domain followed by the SAND (SP100, AIRE, NucP41/P75, and DEAF) domain, plant homeobox (PHD) zinc finger, and bromodomain. The HSR has striking similarities with the caspase recruitment domain (CARD), which mediates homophilic interactions and has been described in proteins involved in apoptosis and inflammatory responses [22]. Previous studies examining SP100 and the autoimmune regulator (AIRE) have shown that the HSR/CARD region is needed for localization to nuclear bodies and for homodimerization [23, 24]. Moreover, SP100, SP110, and SP140 colocalize with promyelocytic leukemia protein (PML) in so-called PML nuclear bodies, which appear as discrete punctate structures in the nucleus [25]. Their heterologous composition and dynamic nature have suggested that these structures can function as regulative depots for nuclear factors [26, 27] and interact with chromatin [28, 29]. Functionally, the SP100 family proteins show various degrees of transcriptional activation and repression [30, 31]. Despite many common traits and links with different pathologies, the exact function of the SP100 family members at the molecular and cellular level remains unknown.

Here, we report a functional characterization of the SP100 family member named SP140L. The comparison of genomic and expressed sequences showed a strong similarity with other family members and indicated that* SP140L* resulted from an unequal meiotic recombination of* SP140* and* SP100* genes that occurred relatively late in the evolution of higher primates. Similarly to other SP100 family members, the highest expression of* SP140L* mRNA can be detected in B cells. We also demonstrate that SP140L protein colocalizes with SP100 and SP140 in the nucleus. In addition, our results show that autoantibodies directed against SP140L are present in the sera of PBC patients.

## 2. Methods

### 2.1. Patients

Sera of 13 patients diagnosed with PBC and all 12 control sera were obtained from the Department of the Internal Medicine, Tartu University Hospital. The use of PBC patient material was approved by the Ethical Committee of Tartu University, and all patients gave their consent for autoantibody studies. Additional nine PBC patients' sera (Table 1, P14–P22) were obtained from a Finnish PBC patient cohort that is described in [32]. All sera were collected before ursodeoxycholic acid treatment. The investigation was conducted according to the principles of the Declaration of Helsinki. The use of human biological material was approved by the Ethical Committee of the Tartu University, and all donors gave their consent for the studies. All healthy donors who donated peripheral blood mononuclear cell (PBMC) material gave written consent for studies. Written consent was also obtained from the majority of the patients allocated for autoantibody studies except from those patients in whom studies have been conducted before year 2000. Only oral consent was obtained from those cases as no written consent was required for the autoantibody studies in Tartu University Hospital. All serum samples originated from Immunology Group Biobank, University of Tartu.

### 2.2. Cloning of* SP140L*,* SP140*,* SP110*, and* SP100A* Coding Sequences

The* SP140L* open reading frame was amplified from the U937 cDNA library with specific primers corresponding to upstream and downstream untranslated regions of* SP140L* mRNA (Table 2). To confirm the correct assignment of the transcription start site, the* SP140L* cDNA 5′ region was amplified from 2 *μ*g of U937 total mRNA with the 5′ RACE (rapid amplification of cDNA ends) methodology (Roche). Primers for the* SP140L*-specific cDNA fragment synthesis and two rounds of amplification are listed in Table 2.

The full-length* SP140L* cDNA was amplified with primers containing restriction sites EcoRI/SalI (Table 2) and cloned into the pM vector (Clontech) to generate a fusion construct with the N-terminal GAL4 DNA-binding domain. EcoRI/NotI sites were used to clone the cDNA into the pcDNA6/myc-His C vector (Invitrogen). However, this construct was very unstable when transformed into bacteria. To overcome the problems with plasmid amplification, the BglII/PaeI fragment containing the* SP140L* cDNA together with the human cytomegalovirus immediate-early promoter and the bovine growth hormone poly(A) signal from pcDNA6/myc-His was ligated with the BamHI/PaeI fragment from the pauxo vector (Icosagen). The latter contained the pUC replication origin and the* araD* gene for selection in* araD*-deficient* E. coli* strains.

The cDNA clones for* SP140* (NM_007237.3) and* SP110* (NM_004509.2) were purchased from OriGene.* SP140* and* SP110* coding sequences were cloned into the EcoRI/SalI sites of pFLAG-CMV-5a (Sigma).* SP100A* coding sequence was amplified from THP1 cell line derived cDNA and cloned into the EcoRI/KpnI sites of pFLAG-CMV-5a expression vector. To perform luciferase immunoprecipitation assays,* SP140L*,* SP140*, and* SP100A* coding sequences were cloned upstream of Gaussia luciferase gene in a mammalian expression vector. The* SP140L* cDNA sequences have been deposited in GenBank under the accession numbers KF419365, KF419366, and KF419367.

### 2.3. Phylogenetic Analysis

The amino acid sequences HSR/CARD, SAND, PHD, and bromodomains of SP140L, SP140, SP100, and SP110 proteins from different primates, including human (*Homo sapiens*), chimpanzee (*Pan troglodytes*), gorilla (*Gorilla gorilla*), orangutan (*Pongo abelii*), macaque (*Macaca mulatta*), tarsier (*Tarsius syrichta*), mouse lemur (*Microcebus murinus*), bushbaby (*Otolemur garnettii*), and marmoset (*Callithrix jacchus*) were aligned with the MUSCLE algorithm [33] and clustered using the online tool at http://www.phylogeny.fr/ [34]. The HSR/CARD, SAND, and Bromo sequences were processed with default settings throughout the analysis. The PHD domain sequences were curated with Gblocks program [35] using less stringent selection criteria.

### 2.4. Cell Sorting

Buffy coats from healthy blood donors were used for cell sorting. Written consent was received from blood donors and the use of peripheral blood material was approved by the Research Ethics Committee of the University of Tartu. PBMCs were isolated by density gradient centrifugation on Ficoll-Paque (GE Healthcare). Cell sorting was performed using MicroBead kits and autoMACS cell sorter from Miltenyi Biotec according to manufacturer's protocols. Plasmacytoid dendritic cells (pDC) were isolated with CD304 (BDCA-4/Neuropilin-1) MicroBead kit, B cells (B) with CD19 MicroBeads, monocytes (MO) with CD14 MicroBeads, CD4+ T cells (CD4+) with CD4 MicroBeads after monocyte depletion, and natural killer (NK) cells with CD56 MicroBeads.

### 2.5. RNA Extraction and Expression Analysis

Total RNA from HeLa, HL60, U937, and THP-1 cell lines and from different subpopulations of PBMCs (pDC, NK, MO, CD40+, and B) was extracted using the TRIzol reagent (Invitrogen). For experiments with interferon stimulation, HL60 and U937 cells were either mock treated or incubated with 250 units/mL of IFN-*α*2a (Miltenyi Biotec) and harvested after 48 h. The RNA was converted to cDNA using an oligo(dT)18 primer and reverse transcriptase (Invitrogen). Reverse transcriptase quantitative PCR (RT-qPCR) reactions were performed on a 7900HT Fast Real-Time PCR System (Applied Biosystems) using SYBR Green I (Eurogentec). All reactions were performed in triplicate and experiments were repeated at least twice. Relative amount of mRNA was calculated using the comparative Ct method (Applied Biosystems): 2^−ΔΔCt^ = 2^−[(Ct_target_−Ct_housekeeping_)  sample−(Ct_target_−Ct_housekeeping_)  control]^, where Ct is the threshold cycle and HPRT or *β*-microglobulins are the housekeeping genes.

### 2.6. Immunoblotting

HEK293 and COS-1 cell lysates containing ectopically expressed SP140L or SP140 proteins were separated by 8% SDS-PAGE and transferred to Immobilon-P PVDF filters (Millipore). Mouse monoclonal anti-Myc 9E10 and anti-FLAG M2 antibodies (1 : 2000 and 1 : 1000, resp.) from Sigma were used to determine the expression of SP140L-Myc and SP140-FLAG. Horse radish peroxidase-conjugated anti-mouse IgG (GE Healthcare) was used as a secondary antibody (1 : 10000). Signals were detected with enhanced chemiluminescence (GE Healthcare) and captured by the ImageQuant-RT ECL image analysis system (GE Healthcare).

### 2.7. Immunofluorescence

For immunofluorescence experiments, the HeLa cells were grown on 8-well chamber slides or on cover slips placed at the bottom of 6-well plates. The cells were transfected with 0.5–0.6 *μ*g DNA on chamber slides and 2–4 *μ*g DNA in 6-well plates. Expression plasmids for pHA-PML and pcDNA3-HA-p300 (gift from N. Shikama, Department of Biomedicine, University of Basel) were used for cotransfections with SP140L-Myc, SP140-FLAG, and SP110-FLAG. Twenty-four hours after transfection, cells were fixed with 3% formaldehyde in phosphate buffered saline (PBS) for 20 min at room temperature (RT). The fixed cells were washed twice with PBS for 5 min followed by permeabilization with 0.5% Triton X-100 in PBS containing 1% normal goat serum for 10 min at RT. All of the following washing and incubation steps were performed with PBS containing 1% normal goat serum at RT. After three 10 min washes, the cells were incubated for 1 h with the following primary antibodies: mouse monoclonal anti-Myc 9E10 and anti-FLAG M2 (1 : 1000) from Sigma, rabbit polyclonal anti-Myc (1 : 5000) from Abcam, rabbit polyclonal anti-HA (1 : 2000) from Santa Cruz Biotechnology, and anti-SP100 from Enzo. After two 10 min washes, the cells were incubated with Alexa Fluor goat anti-mouse 488 or 594 and Alexa Fluor goat anti-rabbit 488 or 594 (1 : 5000) (Invitrogen) secondary antibodies for 1 h. Cells were washed four times for 10 min. DAPI (1 : 2000) was added during the third wash. Slides were mounted with Fluorescent Mounting Medium (DakoCytomation) and examined by the AF6000 LX fluorescence imaging system (Leica Microsystems). The cytofluorogram data and Pearson's correlation coefficients for the overlapping green and red pixel intensities were obtained with the JACoP [36] plugin for ImageJ [37].

### 2.8. Activation Assays

All transfections were performed with ExGen 500* In vitro* Transfection Reagent (MBI Fermentas). For activation assays with GAL4 DNA-binding domain-containing fusion proteins, the cells were grown in 24-well plates and transfected with 0.8–1 *μ*g DNA per well. The DNA mix included pM-SP140L, pM-SP140, or pM-SP110 together with p(GAL4)_3_-tk-Luc reporter plasmid. The cells were harvested and lysed 48 h after transfection. For activation assays with dexamethasone treatment, the cells were grown in 12-well plates and transfected with 2.2 *μ*g DNA per well. The DNA mix included SP140L-Myc, SP140-FLAG, or SP110-FLAG together with glucocorticoid receptor (GR) and MMTV-Luc reporter plasmid. The cells were treated with dexamethasone (1 mM) 24 h later, and the cell lysates were collected 48 h after transfection. Luciferase reporter assays were performed using the Luciferase Assay System (Promega). Luminescence was measured with the Wallac 1420 Victor2 Multilabel Plate Reader (Perkin Elmer). Transfections with the empty pcDNA3.1(−)Myc/His vector (Invitrogen) were used as negative controls.

### 2.9. Detection of Autoantibodies with the Luciferase Immunoprecipitation System (LIPS) and Immunoblotting

For the LIPS assays, HEK293 cells were transfected with Gaussia luciferase-tagged SP100A, SP140, and SP140L expression plasmids by using the TurboFect Transfection Reagent (Thermo Scientific). The samples were harvested and processed after 48 h according to the protocol in [38]. Luminescence was measured with the Victor X5 Multilabel Plate Reader (Perkin Elmer).

## 3. Results

### 3.1. Cloning of Human* SP140L* cDNA

**Figure 1:**
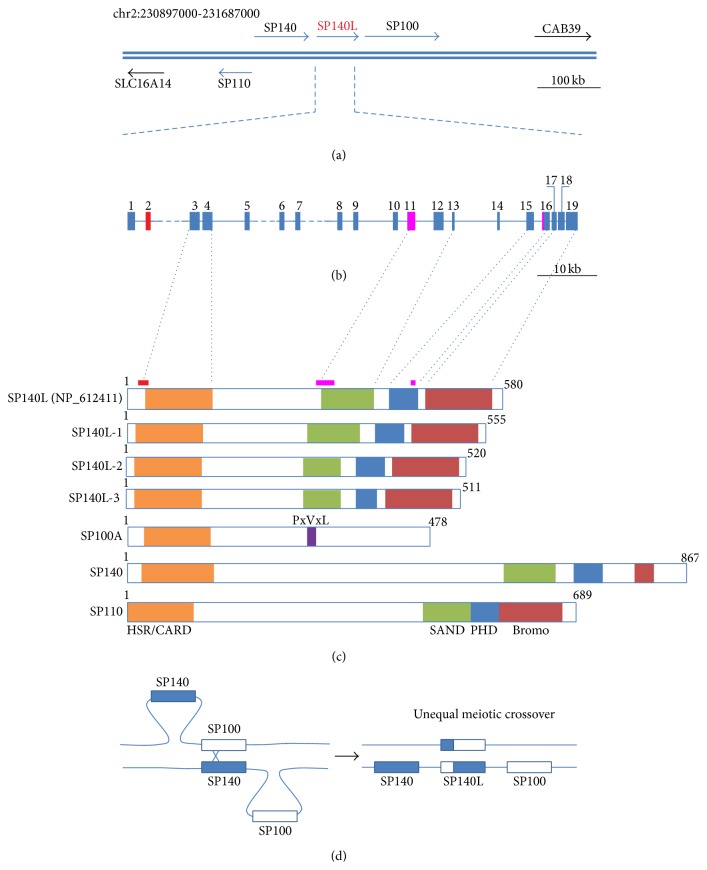
Sequence analysis of the SP100 gene family. (a) Genomic region of human chromosome 2 comprising the SP100 gene family. (b) Genomic structure of the* SP140L* gene. Exons are marked with solid boxes and numbered. Exon 2 (red) is not found in our cDNAs. Exon 11 and a fragment of exon 16 (purple) were spliced in some of our transcripts. (c) Structure of the SP140L protein and its isoforms and comparison to other SP100 family proteins. HSR/CARD (homogenously stained region/caspase recruitment domain), SAND (SP100, AIRE, NucP41/P75, and DEAF), PHD (plant homeodomain type zinc finger), Bromo (bromodomain), and PxVxL (HP1 binding motif). (d) Schematic representation of the putative genomic rearrangements creating the* SP140L* gene.

Previous studies have characterized three SP100 family genes on chromosome 2q37.1:* SP100*,* SP110*, and* SP140* [1–3, 13]. We cloned and sequenced the full-length* SP140L* cDNA from human monocytic U937 cell line (Figure 1(a)). The longest open reading frame was 1668 bp, which lacked the 75 bp exon 2 found in the provisional RefSeq entry NM_138402 (Figure 1(b)). In addition, one splice variant lacked exon 11 and a second variant lacked exon 11 together with 27 bp from exon 16. The cloned* SP140L* cDNA encodes a protein of 555 amino acids and shares a similar protein structure with other SP100 family members (Figure 1(c)). In contrast to other members, the SP140L protein has a shorter sequence between the HSR/CARD and SAND domains.

### 3.2. *SP140L* Has Been Duplicated from* SP100* and* SP140* Genes in Higher Primates

The alignment of the* SP140L* sequence revealed that the* SP140L* gene was formed as a fusion of two neighboring genomic loci in which the first 5 exons that originated from* SP100* and the last 14 exons from* SP140*. This suggested that the* SP140L* gene emerged from a recent duplication event via unequal meiotic crossover (Figure 1(d)). We compared the* SP140*-*SP140L*-*SP100* locus to homologous loci from other mammals and found a structurally similar genomic region among the closely related great apes (chimpanzee, gorilla, and orangutan). The* SP140L* gene was present in the genome of the macaque but not in the marmoset, a representative of New World monkeys.* SP140L* appears to be specific for higher primates as the SP140L gene region is lacking in other mammals, including mouse, rat, dog, cat, cow, and horse.

**Figure 2:**
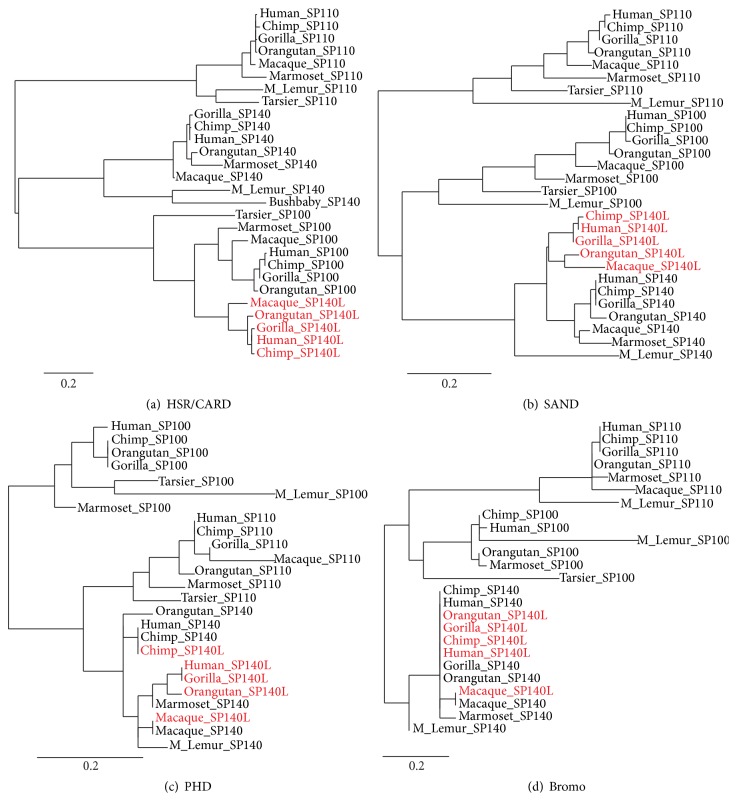
Phylogenetic clustering of SP140L protein domains. (a) HSR/CARD, (b) SAND, (c) PHD, and (d) bromodomain of human, chimpanzee, gorilla, orangutan, and macaque SP140L proteins.

To further support the meiotic crossover event between* SP100* and* SP140*, we did a phylogenetic analysis of HSR/CARD, SAND, PHD finger, and Bromo as the characteristic domains of SP100-related proteins. We included all available sequences of primate origin from the Ensembl database [39] in a multiple sequence alignment and clustering analysis. The phylogenetic tree showed that the HSR/CARD domain of SP140L preferentially clustered with SP100 protein (Figure 2(a)), whereas the SAND, PHD finger, and bromodomain clustered with SP140 (Figures 2(b)–2(d)). Interestingly, the PHD fingers and bromodomains of SP140 and SP140L were highly similar to each other and separate domain clusters could not be distinguished.

### 3.3. *SP140L* Is Interferon-Inducible and Expressed in Immune Cells 

**Figure 3:**
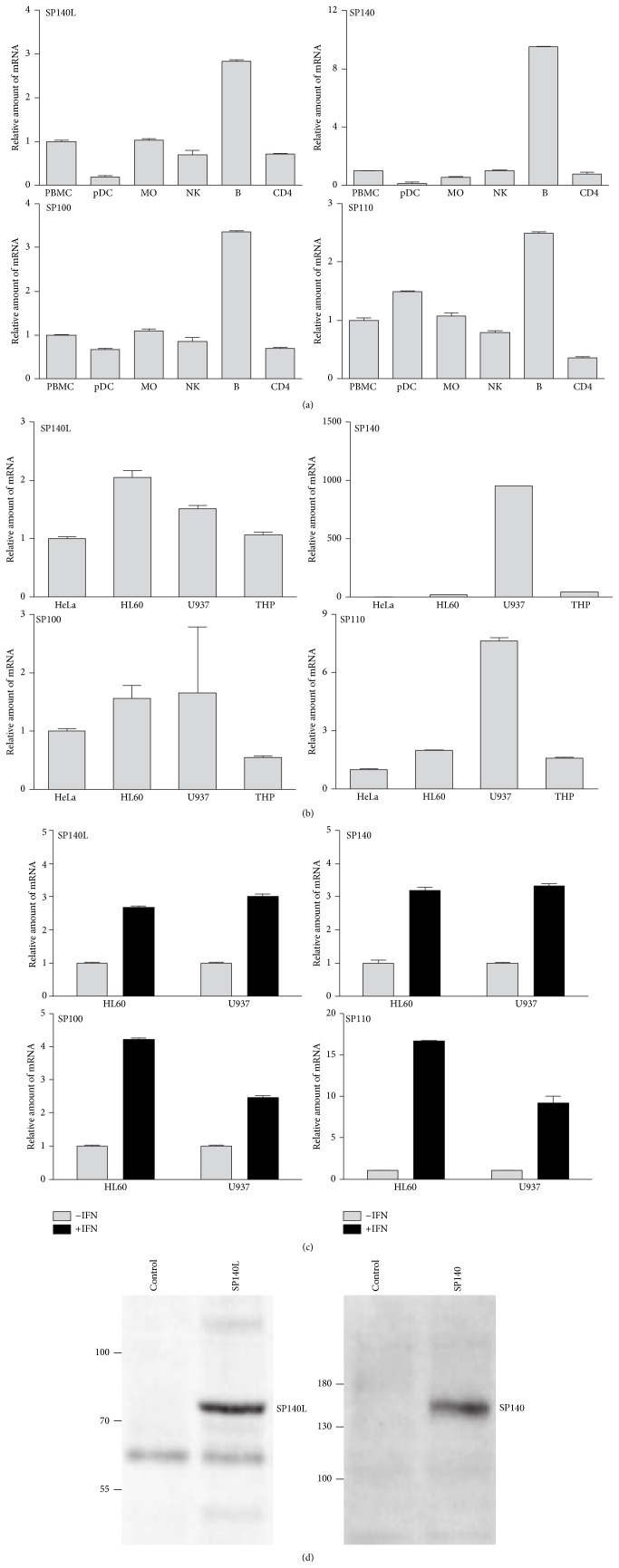
Expression analysis of* SP140L*,* SP140*,* SP100*, and* SP110* in PBMCs and cell lines. (a) The mRNA expression of the SP100 family members in the subpopulations of PBMCs: pDC (plasmacytoid dendritic cells), MO (monocytes), NK (natural killers), B (B cells), and CD4 (CD4+ T cells). (b) The mRNA expression of the SP100 family members in HeLa, HL60, U937, and THP-1 cell lines. (c) The activation of mRNA expression after interferon stimulation in U937 and HL60 cell lines. The mRNA expressions were detected using RT-qPCR. One representative experiment is shown and the error bars show the standard error of the mean (SEM) of the technical replicates. (d) Ectopic expression of SP140L and SP140 proteins in HEK293. The proteins were detected using anti-Myc and anti-FLAG antibodies, respectively.

We noted the specific expression of* SP140L* and other SP100 family members in peripheral blood monocytes, NKs, and T and B cells in the BioGPS database (http://biogps.org/). We therefore studied their expression in PBMCs and in primary sorted cell subsets: dendritic cells, monocytes, NK cells, B cells, and CD4-positive T cells. All SP100 family genes had the highest transcript levels in B cells (Figure 3(a)). We also found the* SP140L* expression in three monocytic cell lines (HL60, U937, and THP1) and in an epithelial (HeLa) cell line, similarly to the* SP100* gene. In contrast, the expression of* SP140* and* SP110* was higher in U937 cells (Figure 3(b)). Earlier studies have shown the upregulation of the gene expression of SP100 family members by interferons [12, 14, 40]. We therefore stimulated U937 and HL60 cells with IFN-*α*2a and observed a 2.5–3-fold increase in* SP140L* mRNA level, indicating that the expression is responsive to interferon stimulation (Figure 3(c)). The interferon-inducible increase was also seen with other SP100 family genes. We then expressed the SP140L-Myc construct in embryonic kidney HEK293 cells and used anti-Myc antibody to detect SP140L with the molecular weight of 75 kDa, which appeared slightly larger than the calculated molecular mass, 64.3 kDa (pI 8.6) (Figure 3(d)). Interestingly, a discrepancy between the predicted and observed molecular masses has also been noticed in immunoblotting with SP140 and SP110 proteins [2, 3]. The difference in expected versus observed mass could be due to posttranslational modifications as several large-scale proteomic studies have identified SP140L to be ubiquitylated [41–45].

### 3.4. SP140L Is Subcellularly Localized to Nuclear Bodies

**Figure 4:**
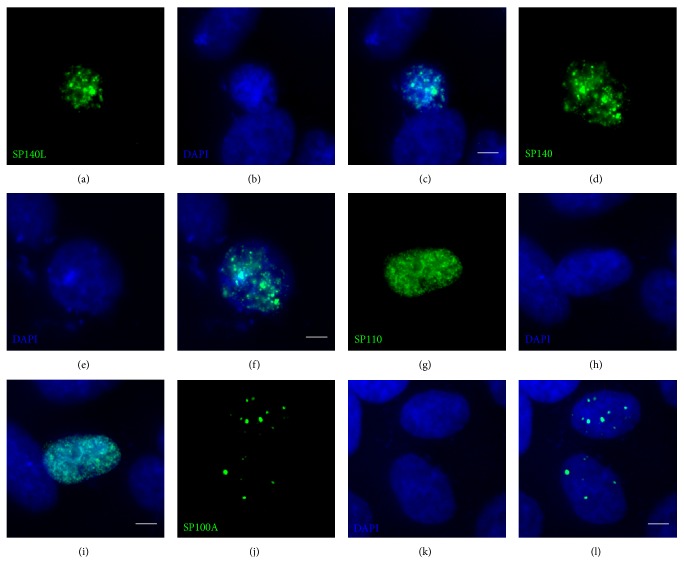
Nuclear localization of the SP140L, SP140, SP110, and SP100A proteins in HeLa cells. SP140L (a–c) and SP140 (d–f) have a highly similar localization pattern in HeLa cell nuclei, which is distinctly different from either SP110 (g–i) or SP100A (j–l). Monoclonal anti-Myc antibody was used to detect SP140L and monoclonal anti-FLAG antibody was used to detect SP140, SP110, and SP100A. Scale bar: 5 *μ*m.

**Figure 5:**
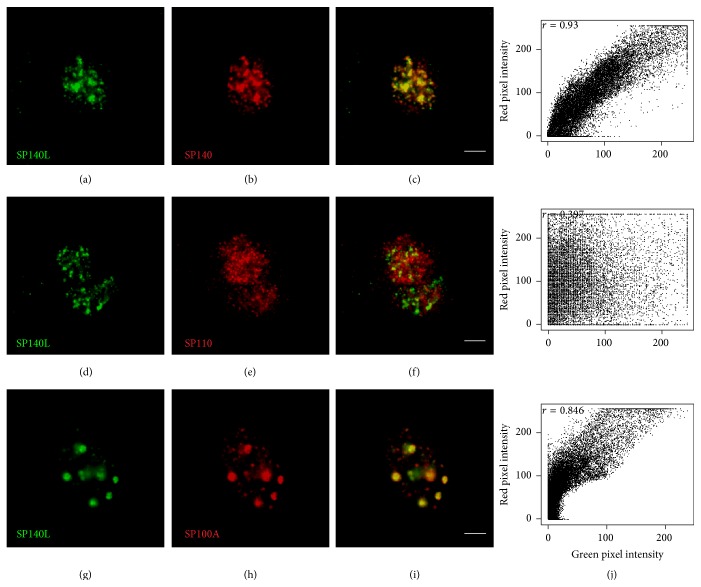
Colocalization of SP140L with other SP100 family members. (a–c) SP140L and SP140. (d–f) SP140L and SP110. (g–i) SP140L and SP100A. (j) Cytofluorograms together with Pearson's correlation coefficients for the overlap of red and green pixel intensities correspond to the images on their left. Polyclonal anti-Myc antibody was used to detect SP140L and monoclonal anti-FLAG antibody was used to detect SP140, SP110, and SP100A. Scale bar: 5 *μ*m.

**Figure 6:**
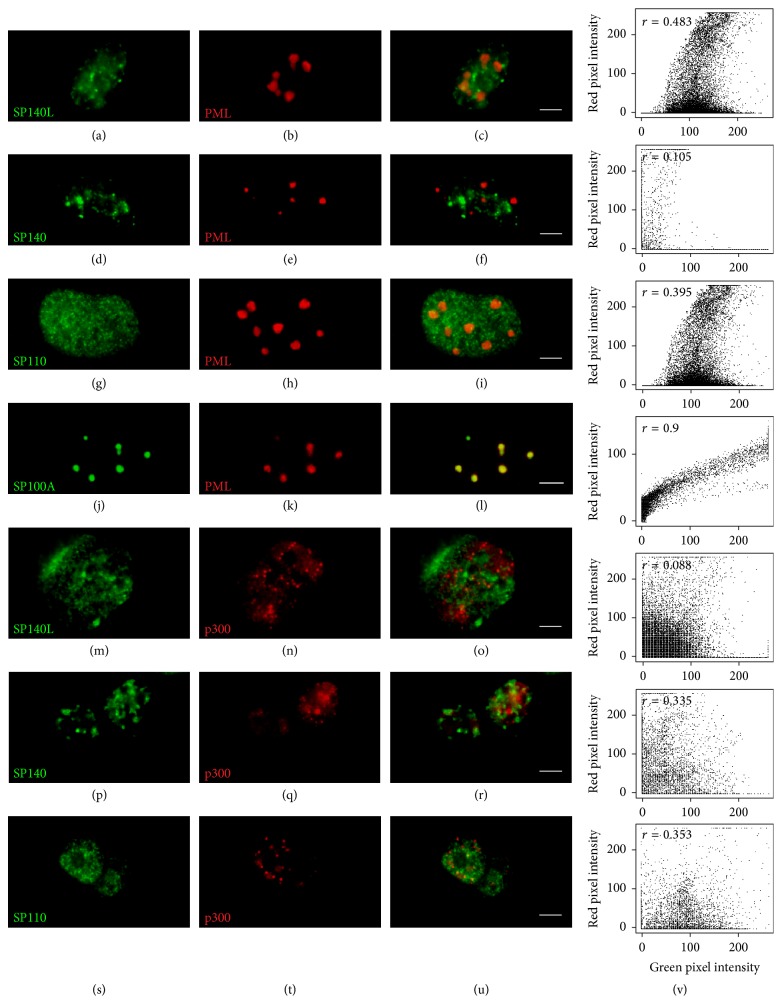
Colocalization of SP140L, SP140, SP110, and SP100A with PML and p300. The nuclear body protein PML does not colocalize with SP140L (a–c), SP140 (d–f), or SP110 (g–i) in HeLa cells. PML colocalizes with its known interaction partner SP100A (j–l). Similarly, the transcriptional coactivator p300 does not colocalize with SP140L (m–o), SP140 (p–r), or SP110 (s–u). Cytofluorograms together with Pearson's correlation coefficients for the overlap of red and green pixel intensities correspond to the images on their left (v). SP140L was detected with monoclonal anti-Myc antibody and anti-FLAG antibody was used to detect SP140, SP110, and SP100A. Polyclonal anti-HA antibody was used to stain HA-PML and HA-p300. Scale bar: 5 *μ*m.

**Figure 7:**
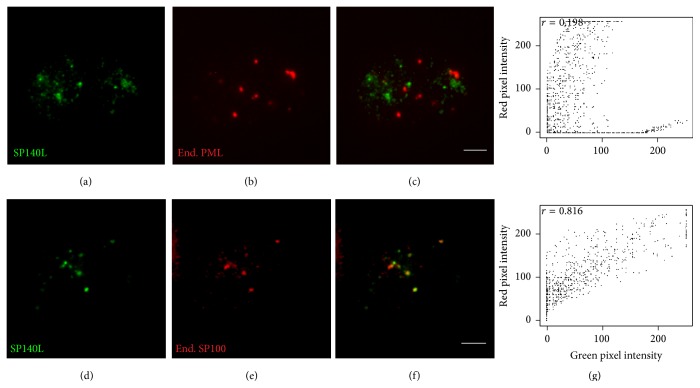
Colocalization of SP140L with endogenous SP100 and PML in HeLa cells. (a–c) Ectopically expressed SP140L does not colocalize with endogenous PML. (d–f) Ectopically expressed SP140L colocalizes with endogenous SP100. (g) Cytofluorograms together with Pearson's correlation coefficients for the overlap of red and green pixel intensities correspond to the images on their left. The proteins were detected with polyclonal anti-Myc (SP140L) and monoclonal anti-SP100 and anti-PML antibodies. Scale bar: 5 *μ*m.

The structural features and similarity with SP100 proteins suggested that SP140L may localize to PML or PML-like bodies. However, the SP140 has been earlier reported to locate into specific subset of nuclear bodies, LYSP100-associated nuclear domains (LANDs) that usually do not overlap with PML bodies and are morphologically different structures [13]. To investigate the subcellular localization, we transfected SP140L-Myc, SP110-FLAG, and SP140-FLAG plasmids into HeLa cells and analyzed the cells by immunofluorescence staining with corresponding antibodies. SP140L was located in nuclear structures that highly resembled the localization pattern of SP140; however, this pattern was distinct from the speckles that were positive for either SP110 or SP100A (Figure 4). We further confirmed localization by cotransfecting SP140L with SP110, SP140, or SP100A and double staining with anti-Myc and anti-FLAG antibodies. SP140 and SP140L showed complete costaining in nuclear bodies (Figures 5(a)–5(c)), which was different from SP110 (Figures 5(d)–5(f)). Interestingly, SP140L lost its fine speckled pattern and colocalized with the SP100A dots (Figures 5(g)–5(i)). These observations were supported by the analysis of correlation of red and green pixel intensities (Figure 5(j)). To study the subcellular location of SP140L protein in relation to PML bodies, we coexpressed SP140L with PML and p300, a transcriptional coactivator found in PML bodies, in HeLa cells and analyzed their colocalization by immunofluorescence. The costaining of SP140L, SP140, or SP110 with PML or p300 protein (Figures 6(a)–6(i) and 6(m)–6(u)) showed weak correlation of signal intensities (Figure 6(v)), whereas SP100A protein colocalized with PML as reported earlier [46] (Figures 6(j)–6(l) and 6(v)). Similarly, costaining of ectopically expressed SP140L and endogenous PML did not reveal colocalization of the two proteins (Figures 7(a)–7(c)). In contrast, a large fraction of endogenous SP100 colocalized with SP140L speckles (Figures 7(d)–7(f)), which was further confirmed by the correlation analysis (Figure 7(g)). We conclude that SP140L and SP140 have identical subcellular localization pattern and are located in nuclear structures that can contain SP100 but are devoid of PML.

### 3.5. SP140L Role in Transcriptional Activation

**Figure 8:**
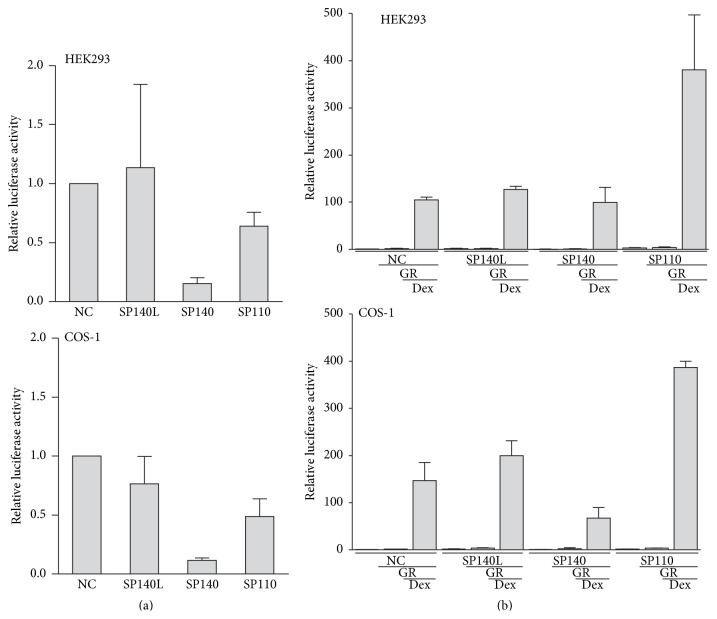
Luciferase reporter gene activation assays with ectopically expressed SP140L, SP140, and SP110 in HEK293 and COS-1. (a) SP140L, SP140, and SP110 fused to GAL4 DNA-binding domain were cotransfected with the luciferase reporter plasmid containing three GAL4 response elements in the promoter. The results represent the mean ± SEM of 3 independent experiments. (b) SP140L, SP140, and SP110 expression plasmids were cotransfected with glucocorticoid receptor (GR) and MMTV-Luc reporter plasmid, and the cells were treated with dexamethasone (Dex). The results represent the mean ± SEM of 3 independent experiments. NC, negative control.

SP100 family proteins, when fused to the GAL4 DNA-binding domain, have been shown to activate or repress the transcription of a reporter from a promoter containing GAL4 response elements [3, 30, 47]. To study whether SP140L acts as a transcriptional regulator, we cloned SP140L, SP140, and SP110 as fusion proteins with a GAL4 DNA-binding domain and transfected them into HEK293 and COS1 cells with a luciferase reporter plasmid that had GAL4 binding sites in the promoter region. We found that SP140L did not have a significant effect on the reporter activity. Contrary to previous data [47], we found that SP140 had a repressive effect on transcriptional activation in the reporter assays (Figure 8(a)).

The SP110 protein has a nuclear receptor (NR) binding motif close to the SAND domain and has been reported to activate transcription from a promoter containing retinoic acid receptor *α* response elements in an all transretinoic acid-dependent manners [3]. To further study the putative function of SP140L in transcriptional regulation, we tested SP140L role in NR-mediated gene expression even though the sequence analysis did not reveal NR boxes in the SP140L protein sequence. We cotransfected SP140L-Myc, SP140-FLAG, and SP110-FLAG together with constructs expressing the GR and the luciferase reporter plasmid with GR response elements inserted into the promoter. As expected, SP110 coactivated GR-mediated transcription of the reporter construct; however, SP140L or SP140 did not enhance the transcriptional activity of GR (Figure 8(b)). Nevertheless, the functional domains of the SP140L protein imply its role in chromatin associated processes, and therefore SP140L could have a role in gene regulation.

### 3.6. SP140L Is an Autoantigen in PBC

**Figure 9:**
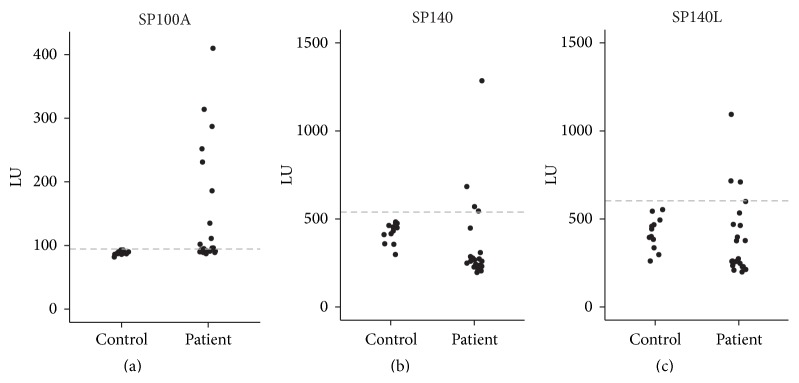
The presence of SP140L-specific autoantibodies in sera from patients with PBC. The LIPS assay detected autoantibodies against SP100A (a), SP140 (b), and SP140L (c) in ten, four, and three patient sera, respectively. Dashed line, 2 standard deviations from the average signal of the control group; LU, light units.

SP100 and SP140 have been identified as autoantigens in PBC [9, 10]. As the presence of SP140 autoantibodies was predominantly found among patients with SP100 autoantibodies, we first screened a cohort of 22 PBC patients and 12 controls (Table 1) for SP100A-specific autoantibodies using the novel luciferase-based immunoprecipitation system (LIPS) [38]. From those, we identified 10 patients with anti-SP100A autoantibodies above the threshold of 2 standard deviations from the control group's average signal (Figure 9(a)). The LIPS assay for SP140 and SP140L proteins found autoantibodies in 4 and 3 PBC patients, respectively (Figures 9(b) and 9(c)).

## 4. Discussion

The genomic order of* SP140-SP140L-SP100* genes seems to be specific for higher primates and phylogenetic analysis indicates the evolvement of* SP140L* gene relatively recently from a common ancestor of Old World monkeys and hominoids through the unequal meiotic crossover of neighboring* SP100* and* SP140* genes. Most likely, the* SP140L* gene duplicated after the divergence of Old World (macaque) from New World monkeys (marmoset; ca. 43 million years ago) and before the separation from hominoids (human and great apes; ca. 30 million years ago) [48].

The expression of* SP140L* is similar to other SP100 family genes, with the highest expression in CD19-positive B cells. According to BioGPS,* SP140L* is expressed in CD19-positive B cells, CD4- and CD8-positive T cells, and NK cells. Our results confirm the expression of* SP140L* in immune cells and show that, like other SP100 family genes,* SP140L* expression is upregulated by interferon stimulation.

The SP140L protein shares the characteristic protein domains with other SP100 family proteins and with AIRE, a transcriptional regulator that interacts with chromatin [49] to activate the thymic ectopic expression of self-antigens [50]. The HSR/CARD domain in SP100 proteins and in AIRE is needed for homodimerization and/or nuclear body localization [3, 24, 51]. The SAND domain, present in SP100B [52] and GMEB1 [53] proteins, binds to unmethylated CpG DNA sequences [54]. The PHD fingers represent a distinct group of zinc fingers recognizing H3K4me0 or H3K4me3 [55, 56]. However, the structure of the SP140 PHD domain, which is highly similar to SP140L PHD domain, renders it incapable of binding to histone H3 N-terminal tail, and the* cis-trans* peptidyl-prolyl isomerization occurring at the Thr726-P727 bond further affects interactions with potential partners [57]. The bromodomain possesses high binding affinity towards acetylated histones [58], although its role among the SP100 family members has not been established. It is plausible that the closely positioned PHD and bromodomain cooperatively determine the binding of the SP100 family members to their interaction partners.

Although the structural domains suggest SP140L to function in transcriptional processes, we did not observe either SP140L-dependent activation of the luciferase reporter gene or coactivation by SP140L and a nuclear hormone receptor that has been reported with SP110 [3]. The transactivation assays, however, are limited to the transcriptional regulation of ectopic plasmids and do not exclude the role of SP140L in epigenetic control of transcription on native chromatin level. SP140L colocalized with SP140 in nuclear structures that are distinct from either ectopically expressed or endogenous PML containing structures. Interestingly, SP140L colocalized with ectopic and endogenous SP100, which is tightly associated to PML nuclear bodies [46]. The colocalization of SP140L and SP100 could reflect heterooligomerization mediated by their highly similar HSR/CARD domains, while the other protein domains, for example, SAND, PHD, or Bromo, may sequester SP140L to complexes that are largely devoid of PML. Seeler et al. [59] describe the SP100C isoform that contains the SAND, PHD, and bromodomains, similarly to SP140L, SP140, and SP110. They find that SP100C colocalizes only with a subset of PML nuclear bodies and displays an altered localization pattern most likely due to an expanded repertoire of interactions through its additional functional domains. Moreover, heterogenic patterning of nuclear bodies with variation between specific cell types has been reported [25]. SP140 has been shown to localize in a separate subset of nuclear bodies named LANDs [13, 60], yet, in some cells, the SP140-positive nuclear structures have been reported to contain PML and SP100 [3, 47].

Finally, we report that SP140L is a novel autoantigen in PBC patients. The prevalence of anti-SP100, anti-SP140, and anti-PML autoantibodies among PBC patients has been reported to be 15–30% [10, 61]. These autoantibodies are most frequently found in PBC patients negative for antimitochondrial antibodies, a well-established serological marker for the disease [62–64]. In 90% of cases, the anti-SP100 and anti-SP140 antibodies coincide in patients [10]. In our limited screening for autoreactivity, we found ten PBC patients with SP100-specific autoantibodies in their sera, while three and four patients tested positive for SP140L and SP140 autoantibodies, respectively. The possibility of antibody cross-reactivity due to the high similarity of SP140L protein domains to SP100 and SP140, however, requires further assessment by epitope mapping. The autoantibody reactivity to several nuclear body proteins suggests that these structures themselves may trigger autoantibodies in PBC. The activation of the autoimmune reaction might be associated with antiviral responses as the expression of all four proteins, including SP140L, is upregulated by interferons, and a possible role of microorganisms as triggers of PBC has been previously suggested [65].

## 5. Conclusions

The present study describes the SP100 gene family member* SP140L* and suggests that the* SP140L* gene is a result of evolutionarily recent genomic rearrangements in higher primates. SP140L shares many features with its neighboring SP100 family members SP100 and SP140.* SP140L* gene expression is enhanced in the presence of interferons and SP140L protein is localized in nuclear bodies. In addition, we describe SP140L protein as a novel autoantigen in PBC patients.

## Figures and Tables

**Table 1:** Clinical and autoantibody data of studied 22 PBC patients.

Patient	Age	Stage	AMA	ANA	SP100 (LIPS)	SP140 (LIPS)	SP140L (LIPS)
P1	50	III	1 : 100	—	+	+	—
P2	51	II	—	1 : 100	+	+	+
P3	54	II	1 : 320	—	—	—	—
P4	63	IV	1 : 100	—	+	—	—
P5	54	IV	1 : 40	—	—	—	—
P6	44	III	1 : 320	—	+	—	—
P7	88	III	1 : 320	—	—	+	—
P8	43	II	1 : 100	—	—	—	—
P9	63	IV	1 : 160	—	—	—	—
P10	65	III	1 : 100	—	—	—	—
P11	66	II	1 : 100	1 : 10	—	—	—
P12	42	III	1 : 100	—	—	—	—
P13	61	II	1 : 100	—	+	+	+
P14	—	IV	1 : 8000	—	+	—	—
P15	79	III	1 : 4000	—	+	—	+
P16	64	III	1 : 8000	—	+	—	—
P17	59	II	1 : 8000	—	—	—	—
P18	70	II	1 : 1000	—	+	—	—
P19	49	IV	1 : 500	—	+	—	—
P20	60	I	1 : 8000	—	—	—	—
P21	—	II	—	—	—	—	—
P22	72	III	1 : 200	—	—	—	—

PBC staging was performed by liver biopsy sample histology. Indirect immunofluorescence on rodent tissue slides was used for AMA (antimitochondrial antibodies) and ANA (antinuclear antibodies) detection at indicated serum dilutions. The LIPS assay is described in Methods.

**Table 2:** Primers used in the study.

Name	Application	5′ → 3′
SP140L_cDNA F1	cDNA amplification	CAAAGCCGATGGCCGGTGGGGGCA
SP140L_cDNA R1		CAGTAGCATATTTCCTGAAGGCC

SP140L_RACE R1	5′ RACE	CAGCTGGCCATCGGCTTTGC
SP140L_RACE R2		TGGTTGTTTTTCTGCGTTCTCG

SP140L F1	Cloning	TTTGAATTCATGGCAGGTGGGGGCAG
SP140L R1		TTTGTCGACTCAACTGTTCCCATTTGTTTC
SP140L R2		TTTGCGGCCGCACTGTTCCCATTTGTTTC
SP140L R3		TTTGGTACCACTGTTCCCATTTG

SP140 F1	Cloning	TTTGAATTCATGGCCCAGCAGGGCCA
SP140 R1		AAAGTCGACTCAATTGTTCCCATTTGTTTCC
SP140 R2		TTTGTCGACATTGTTCCCATTTGTTTCCTG
SP140 R3		TTTGGTACCATTGTTCCCATTTGTTTC

SP110 F1	Cloning	TTTGAATTCATGTTCACCATGACAAGAGC
SP110 R1		AAAGTCGACTCAAGGAAGAGTCCAGAA
SP110 R2		TTTGTCGACAGGAAGAGTCCAGAAACC

SP100A F	Cloning	TTTGAATTCATGGCAGGTGGGGGCGGCGA
SP100A R		TTTGGTACCATCTTCTTTACCTGACCCTCTTCTTAGG

SP140L_QPCR F1	Expression analysis	TGGAAGCACTGTTCAGCGAGGT
SP140L_QPCR R1		TGATGTCAGGCCTCTCTTCCCT

SP140_QPCR F1	Expression analysis	CCAGGTGGGGGAGTGTCCTGT
SP140_QPCR R1		TCTCCCCTGGTGCTGTGCTGT

SP110_QPCR F1	Expression analysis	CCATACCCCACTGGCGCTGC
SP110_QPCR R1		GTCAGATGGGCTGGGCGAC

SP100_QPCR F1	Expression analysis	GGCTGAGCCAACAGAGTCCTGCG
SP100_QPCR R1		TCCACCAGTCGCACAGAACAGGA

B2M_QPCR F1	Expression analysis	TGCTCGCGCTACTCTCTCT
B2M_QPCR R1		TCCATTCTCTGCTGGATGAC

HPRT_QPCR F1	Expression analysis	GACTTTGCTTTCCTTGGTCAGG
HPRT_QPCR R1		AGTCTGGCTTATATCCAACA CTTCG
